# Lactic Acid Production from Pretreated Hydrolysates of Corn Stover by a Newly Developed *Bacillus coagulans* Strain

**DOI:** 10.1371/journal.pone.0149101

**Published:** 2016-02-10

**Authors:** Ting Jiang, Hui Qiao, Zhaojuan Zheng, Qiulu Chu, Xin Li, Qiang Yong, Jia Ouyang

**Affiliations:** 1 College of Chemical Engineering, Nanjing Forestry University, Nanjing, 210037, People’s Republic of China; 2 Key Laboratory of Forest Genetics and Biotechnology of the Ministry of Education, Nanjing Forestry University, Nanjing, 210037, People’s Republic of China; National Renewable Energy Lab, UNITED STATES

## Abstract

An inhibitor-tolerance strain, *Bacillus coagulans* GKN316, was developed through atmospheric and room temperature plasma (ARTP) mutation and evolution experiment in condensed dilute-acid hydrolysate (CDH) of corn stover. The fermentabilities of other hydrolysates with *B*. *coagulans* GKN316 and the parental strain *B*. *coagulans* NL01 were assessed. When using condensed acid-catalyzed steam-exploded hydrolysate (CASEH), condensed acid-catalyzed liquid hot water hydrolysate (CALH) and condensed acid-catalyzed sulfite hydrolysate (CASH) as substrates, the concentration of lactic acid reached 45.39, 16.83, and 18.71 g/L by *B*. *coagulans* GKN316, respectively. But for *B*. *coagulans* NL01, only CASEH could be directly fermented to produce 15.47 g/L lactic acid. The individual inhibitory effect of furfural, 5-hydroxymethylfurfural (HMF), vanillin, syringaldehyde and *p*-hydroxybenzaldehyde (pHBal) on xylose utilization by *B*. *coagulans* GKN316 was also studied. The strain *B*. *coagulans* GKN316 could effectively convert these toxic inhibitors to the less toxic corresponding alcohols *in situ*. These results suggested that *B*. *coagulans* GKN316 was well suited to production of lactic acid from undetoxified lignocellulosic hydrolysates.

## Introduction

Lignocellulosic biomass, especially agricultural and forest residues, is a potentially low-cost renewable resource of sugars for fermentation [1,2]. Its utilization could not only decrease the demand for petroleum and food raw materials but also might alleviate the environmental pressure concerning CO_2_ emissions from fossil fuels. In China, corn stover is an agricultural residue that could be used for the production of biofuel and green chemicals [3]. However, the bioconversion and exploitation of this feedstock still face several technical obstacles at this time. Lignocelluloses are a matrix of cross-linked polysaccharide networks, which mainly consists cellulose, hemicelluloses and lignin [4]. The efficient utilization of pentose, mainly xylose, from hemicelluloses still remains a challenge for the economic feasibility of bioconversion [5,6]. Moreover, during most of the pretreatment methods, along with a great amount of pentose from the hemicelluloses liberated into prehydrolysate, a number of toxic compound which are inhibitory to microbial fermentation, were stimulatingly formed due to the severe condition [7]. The existence of these inhibitory compounds increases the degree of difficulty for the microorganism to undergo xylose fermentation [8,9].

Numerous studies discussed the generation of various inhibitors and their effects on the fermentation yield and productivity of yeasts [10,11]. As reported, the components of the inhibitory compounds vary greatly with the pretreatment method and the raw material used. These inhibitory compounds were generally divided into three major groups: weak acids (i.e. formic, acetic, and levulinic acid); furan derivatives (furfural and HMF); and phenolic compounds [12,13]. Among these inhibitory compounds, phenolic compounds, especially low-molecular-weight phenols, have a significant inhibitory effect and are generally more toxic than furfural and HMF for the microorganism [13,14]. However, due to their low concentration and complexity, it is still difficult to properly evaluate the toxic nature of the hydrolysates. To tackle the problem of toxicity, a number of physical, chemical and biological detoxification methods have been developed to overcome the inhibitory effects [7,12,15]. At the same time, these additional treatments must add the cost and complexity of the detoxification process [16]. The search for a fermenting organism that can both utilize xylose and tolerate these compounds for industrial processing offers a promising alternative that avoids the need for separate detoxification steps. The adaptation of microorganisms to the lignocellulosic hydrolysate, possibly after inducing variation by mutagenesis, serves as an alternative option that might improve the fermentation processes and increase its economic feasibility [7,17].

Moderately thermophilic *Bacillus coagulans* are ideal organisms for the industrial manufacture of lactic acid. Some strains, such as 36D1, MXL-9, and C106, ferment both glucose and xylose to optically pure L-lactic acid at temperatures above 50°C under anaerobic conditions [18–20]. In our previous study, a wild-type *B*. *coagulans* NL01 demonstrated good potential for L-lactic acid production using renewable resources [21,22]. Here we aim to develop a derivative strain from NL01 with a broadly improved tolerance against toxic hydrolysates. *B*. *coagulans* GKN316 was screened and obtained by atmospheric and room temperature plasma (ARTP) mutation and a directed adaptation using hemicellulose hydrolysate from corn stover treated with dilute sulfite acid. Then, the fermentation performance of *B*. *coagulans* GKN316 and NL01 using other pretreatment hydrolysates were compared. Finally, the inhibitory effect of furan derivatives and phenolic compounds on the growth and fermentation of *B*. *coagulans* GKN316 were investigated and the conversion products are presented here.

## Materials and Methods

### Materials

Weak acids, furan aldehydes and phenolic compounds, as well as the other chemical standards, were purchased from Sigma chemicals. The chemicals used in microbiological culture media were purchased from Sinochem or Fluka Chemical and were of analytical grade. Corn steep liquor was from Shandong Kangyuan Biotechnology Co. (Heze, China). Corn stover was obtained from Lian Yungang in China.

### Preparation of the various hydrolysates

Corn stover was cleaned, chopped and screened to a size of 0.2–0.8 mm for the subsequent pretreatment. Dilute-acid hydrolysate (DH), with the biomass at a solid loading rate of 10%, was prepared at 160°C with 2% (w/v) H_2_SO_4_ and the residence time was 60 min. Acid-catalyzed steam-exploded hydrolysate (ASEH) was prepared with 1.29% (w/v) H_2_SO_4_ at 0.8 MPa (gauge pressure) and 175°C for 5 min. Liquid hot water hydrolysate (LH) was prepared in a laboratory-scale stirred autoclave with water at a 1/10 solid/liquid ratio. The pretreatment condition was at 180°C and 500 rpm for 40 min. Sulfite hydrolysate (SH) was prepared by immersing the corn stover in 4% (w/v) Mg(HSO_3_)_2_ at 160°C for 60 min, and the ratio of the solid/liquid was 1/6. When the pretreatment was finished, the reactor was immediately cooled down using cooling water.

Subsequently, the four prehydrolysates were collected by filtration. After adjusting to a pH of 6.0 with Ca(OH)_2_ and filteration, the various hydrolysates were concentrated in a BÜCHI rotary evaporator R-00 from BüCHI Shanghai Trading LLC (Shanghai, China) at 60°C and 160 mbar. The four condensed hydroysates were used for the lactic acid fermentation and were called condensed dilute-acid hydrolysate (CDH), condensed acid-catalyzed steam-exploded hydrolysate (CASEH), condensed liquid hot hydrolysate (CLH), and condensed sulfite hydrolysate (CSH), respectively.

### Microorganisms and medium

*B*. *coagulans* NL01 (CCTCC NO. M2011468) and *B*. *coagulans* GKN316 (a mutant derived from *B*. *coagulans* NL01) were used in this study. The medium used for growing *B*. *coagulans* contained the following (in grams per liter): xylose, 20; yeast extract (YE), 2; corn steep, 2.5; NH_4_Cl, 1; MgSO_4_, 0.2 and CaCO_3_, 10. The adaptation medium was prepared by adding xylose to 20 g/L and glucose to 4 g/L based on the growth medium in 25% to 80% (v/v) filtrate of CDH (Table 1). The culture medium used for fermentation was as follows (in grams per liter): yeast extract (YE), 2.5; corn steep 1.2; (NH_4_)_2_SO_4_, 3; KH_2_PO_4_, 0.22; MgSO_4_**·**7H_2_O, 0.4; MnSO_4_**·**H_2_O, 0.03; FeSO_4_**·**7H_2_O, 0.03; and different carbon sources with about half as much CaCO_3_ as the total sugars. The type and concentration of the carbon sources varied in the different fermentation experiments. The selection agar plates were made with the adaptation medium with 80% CDH and 16 g/L agar.

**Table 1:** Major composition of condensed dilute-acid hydrolysate (CDH), condensed acid-catalyzed steam-exploded hydrolysate (CASEH), acid-catalyzed liquid hot water hydrolysate (CALH) and condensed acid-catalyzed sulfite hydrolysate (CASH).

Components	CDH	CASEH	CALH	CASH
**Glucose (g/L)**	4.76 ± 0.16	7.41 ± 0.27	2.25 ± 0.03	3.80 ± 0.24
**Xylose (g/L)**	23.87 ± 0.22	40.19 ± 0.41	16.37 ± 0.12	20.56 ± 0.07
**Arabinose (g/L)**	2.31 ± 0.09	6.95 ± 0.15	2.09 ± 0.07	0.621 ± 0.26
**Formic acid (g/L)**	1.33 ± 0.15	0.72 ± 0.09	1.81 ± 0.03	0
**Acetic acid (g/L)**	2.98 ± 0.09	2.0 ± 0.06	4.67 ± 0.25	11.48 ± 0.08
**Levulinic acid (g/L)**	1.10 ± 0.13	0.28 ± 0.05	0.19 ± 0.06	0
**Furfural (g/L)**	0.05 ± 0.01	0	0	0
**HMF (g/L)**	1.45 ± 0.18	0.39 ± 0.13	0.21 ± 0.04	0.55 ± 0.10
**TPC (g/L)**	2.41 ± 0.11	3.07± 0.03	2.96 ± 0.08	23.43 ± 1.71
**Vanillin (mg/L)**	25.62 ± 0.37	44.19 ± 0.20	58.78 ± 2.13	172.64 ± 1.14
**syringaldehyde (mg/L)**	13.89 ± 0.90	21.53 ± 0.61	10.35 ± 0.17	168.43 ± 2.14
***p*-hydroxybenzaldehyde (mg/L)**	11.43 ± 0.84	17.18 ± 1.01	44.65 ± 1.25	41.34 ± 0.62

Values are the average ± SD of three separate experiments.

### Evolutionary engineering and selection of mutants

Helium-based atmospheric and room temperature plasma (ARTP) was used to create mutants of *B*. *coagulans* NL01. For the mutation, 10 μL of the culture (OD_600_ = 1.0) was daubed onto a sterilized stainless steel plate and dried in sterile air for a few minutes. As the metal plate with the bacterial cells was treated for 20 s by the helium plasma jet, the plate was washed with 1 mL of sterilized saline solution. Then, 200 μL of the eluted solution was inoculated into a 100-mL shake flask with 25 mL of the adaptation medium of 25% CDH.

For evolutionary engineering, the mutant strains were first grown in an adaptation medium containing low concentrations of CDH at 50°C and 150 rpm. Once logarithmic growth was reached, the surviving cells were transferred into another shake flask containing fresh medium. When the adapted cultures became stable, the inoculum level was gradually reduced from the inoculation ration of 20% to 2%. When the adapted culture was established, the concentration of CDH was increased in the subsequent shake flask. This reiterative process was sustained until a desirable tolerance level to CDH was reached. After adaptation, the mutant cells were spread over the selective agar plates to isolate the high inhibitor-tolerance mutants.

### Fermentation experiments

*B*. *coagulans* was grown at 50°C on agar slants for 16 h. To prepare the inocula the organism was transferred by a loop from the slants to a liquid growth medium and was inoculated by agitation at 150 rpm for 12 h at 50°C in aerobic shake flasks. This inoculate culture was used to provide a 10% (v/v) inocula for each fermentation experiment. Fermentation medium (50 mL) was added to each flask with varying types of carbon sources at 50°C, at a pH of 7.2 and at 150 rpm under oxygen-limited conditions. The samples were collected periodically for determining the cell mass, the residual substrates, and the lactic acid and inhibitor concentrations as described below. All of the fermentation experiments were performed in triplicate.

### Analytic methods

The sugar, lactic acid, formic acid, acetic acid, levulinic acid, furfural and HMF concentrations of the samples were analyzed by an Agilent HPLC 1260 system using a 300-mm Aminex HPX87H column (Bio-Rad Laboratories, Inc. Hercules, CA) and a refractive index detector. The mobile phase was 5 mM H_2_SO_4_ at a flow rate of 0.6 mL/min, and the column temperature was maintained at 65°C.

For the determination of the phenolic compounds (*p*-hydroxybenzoic acid, vanillic acid, syringic acid, *p*-hydroxybenzaldehyde (pHBal), vanillin and syringaldehyde), reversed-phase HPLC was carried out on an Agilent 1260 unit with a DAD detector using a 250-mm Zorbax Eclipse XDB-C18 column. The mobile phase was acetonitrile (100%)–water (containing 1.5% acetic acid) at 30°C at a flow rate of 0.8 mL/min, and the detection wavelengths were 254 nm and 280 nm. All of the samples were centrifuged at 10,000 rpm for 5 min and filtered through 0.22-μm syringe filters.

GC was used to analyze the metabolism of the inhibitor supplements in the fermented test medium. The samples were centrifuged at 10,000 rpm for 10 min. The supernatant was collected, saturated with NaCl, and extracted three times with the same volume of anhydrous ethyl acetate. Then, the combined ethyl acetate extracts were dried over MgSO_4_, and centrifuged to remove the solid. Trifluorobis(trimethylsiyl)–acetamide (BSTFA) (0.4 mL) and pyridine (0.1 mL) were added to 0.5 mL of the dried samples (except furfural) for the silylated reactions at 70°C for 30 min. GC—MS analysis was carried out on a Thermo Finnigan TRACE DSQ GC—MS instrument with an Agilent 0.25 mm×30 m DB-5 fused silica capillary column with a film thickness of 0.25 μm. Helium was used as the carrier gas at a constant column flow rate of 1 mL/min with injector and ion-source temperature of 250°C. The column temperature was programmed as follows: 50°C (2 min), increasing to 280°C (held for 5 min) at a rate of 10°C/min. The mass detector was taken at 70 eV, and the scanning mass range was from 33 to 450 amu. The identification of the hydrolysates was achieved by comparing their mass fragments with the National Institute of Standards and Technology (NIST) mass spectral library.

The total phenolic content in the samples was estimated using the Folin—Ciocalteu’s method [23]. One milliliter of a 0.6 M Folin—Ciocalteu reagent was added to either 1 mL of the sample or 1 mL of the standard solutions (gallic acid). Then, 3 mL 6% sodium carbonate solution was added, and 5 mL H_2_O was added to yield a total test solution volume of 10 mL. After being mixed and held at 30°C for 2 hours, the solution was transferred into a cuvette and measured at 745 nm using an Ultrospec TM 2100 pro (GE Healthcare, USA). The total phenolic content in the samples was calculated based on linear regression of the standard.

Productivity was calculated as the concentration of lactic acid produced per liter divided by the fermentation time (h) and is expressed as g/L/h. Lactic acid yield was calculated as the percentage of determined lactic acid to the theoretically calculated lactic acid produced from the consumed glucose, xylose and arabinose (1 g lactic acid/ g glucose or g xylose or g arabinose).

## Results and Discussion

### Screening of the high inhibitor-tolerance mutant strain

Previously, we showed that *B*. *coagulans* NL01 produces 18.2 g/L lactic acid from a steam-exploded prehydrolysate (which contained 25.45 g/L of reducing sugar) [24], but this strain was inhibited when the hydrolysate was at a higher concentration. So, in the present study, a coupled ARTP mutagenesis technique and a directed adaptation method for *B*. *coagulans* NL01 were applied to obtain a more inhibitor-tolerant strain.

After the ARTP mutation of *B*. *coagulans* NL01, 200 μL of the mutant cells were transferred into a fresh medium broth to initiate a long-term evolutionary adaptation experiment. The surviving strains were challenged with the condensed dilute-acid hydrolysate (CDH) at concentrations increasing from 25% to 80%. To monitor the adaptation process, the population growth phenotypes and the sugar-lactic acid content were measured after every 6-h transfer into fresh medium. The initial sugar concentrations were maintained at 4 g/L glucose and 20 g/L xylose. After approximately 50 population transfers, the cells were collected and spread over selective agar plates to screen the high inhibitor-tolerance mutants. After cultivation at 50°C for 48 h, approximately 60 colonies showed large and transparent halos on the selective agar plates and were chosen for fermentation with 80% CDH (details not shown). Among them, a new *B*. *coagulans* strain named GKN316 demonstrates the highest level of tolerance to acid hydrolysate.

### Fermentation behavior of *B*. *coagulans* GKN316 on CDH

**Fig 1:**
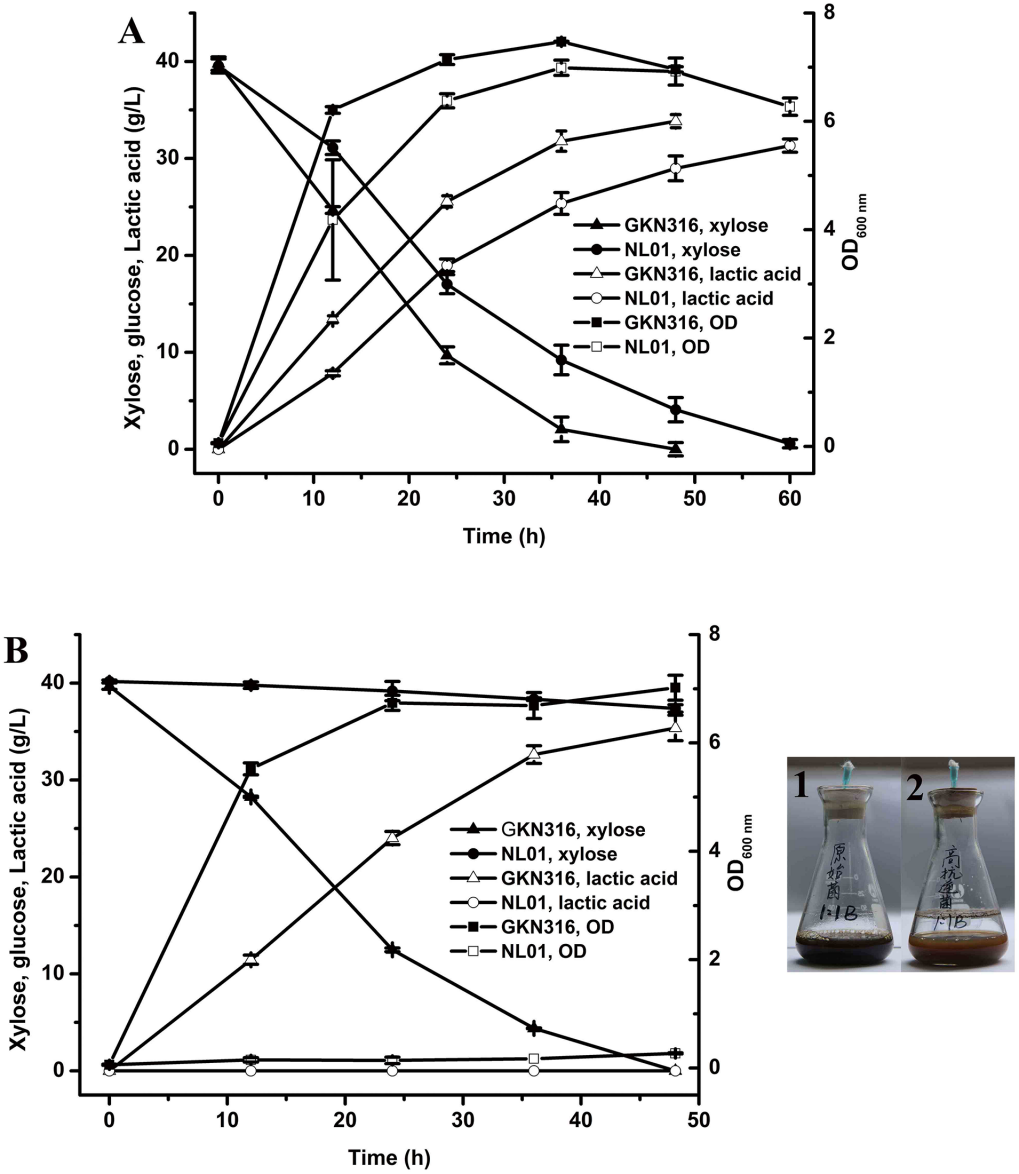
Comparison of lactic acid production and cell growth by using *Bacillus coagulans* NL01 and *Bacillus coagulans* GKN316 with different concentrations of condensed dilute-acid hydrolysate (CDH). (**A**) 25% hydrolysate; (**B**) 50% hydrolysate. The color of hydrolysate culture of *B*. *coagulans* NL01 (**B1**) and GKN316 (**B2**) after 24-h fermentation. Values are the average ± SD of three separate experiments.

To determine whether GKN316 had an advantage over the parental strain NL01 using acid prehydrolysate, the cell growth and lactic acid production of both strains were compared using 25% or 50% CDH (Fig 1). Before the fermentation, either 25% or 50% CDH was supplemented with xylose to reach 40 g/L. As shown, the strain GKN316 reached the stationary phase in 12 h in the xylose medium containing 25% CDH, which was slightly faster than the parental strain. However, under the challenge of 50% CDH, only the strain *B*. *coagulans* GKN316 showed a quick cell growth during the first 24 h. By contrast, the parental strain NL01responded with an extended lag phase, and cell growth did not recover at the end of fermentation (48 h). A number of previous adaptation studies showed a qualitative change in cell response to the presence of the inhibitors compared with the parental strains [9,25,26]. Hence, the growth of GKN316 at the higher acid hydrolysate concentration indicated that it had a better fermentation performance in the treated hydrolysates compared to the control strain.

As shown in Fig 1A and 1B, the new strain had a better lactic acid yield and xylose consumption rate than the parental strain. The GKN316 strain exhibited a maximum lactic acid titer of 33.84 g/L after 48 h of fermentation at a 25% CDH concentration, while the lactic acid production of *B*. *coagulans* NL01 reached its maximum value of 31.32 g/L at 60 h. As for the fermentation of 50% CDH, the parental strain NL01 did not show significant xylose consumption, and no lactic acid production was detectable. However, for the GKN316 strain, 40 g/L xylose consumption was completed and the maximum lactic acid production (35.37 g/L) was reached 48 h after fermentation. In this case, the volumetric lactic acid productivity of the strain *B*. *coagulans* GKN316 was 0.91 g/L/h in the first 36 h. Our results suggested that *B*. *coagulans* GKN316 increased the ability to withstand and grow in lignocellulosic hydrolysates. Similar positive results were obtained with the adaptation of *Saccharomyces cerevisiae* to dilute sulfurous acid spruce hydrolysate [27] and *Clostridium beijerinckii* to dilute sulfuric acid corn fiber hydrolysate [28]. These adapted, more tolerant strains showed no significant delay in cell growth and sugar consumption. Thus, they produced a normal yield even in the presence of inhibitors. Additionally, compared with the color of fermentation culture by the parental strain NL01, the color of fermentation culture by GKN316 became bleached, which might be due to the degradation of some of the colored compounds, especially the phenolic compounds.

### Fermentation behavior of the evolved strain on other hydrolysates

**Fig 2:**
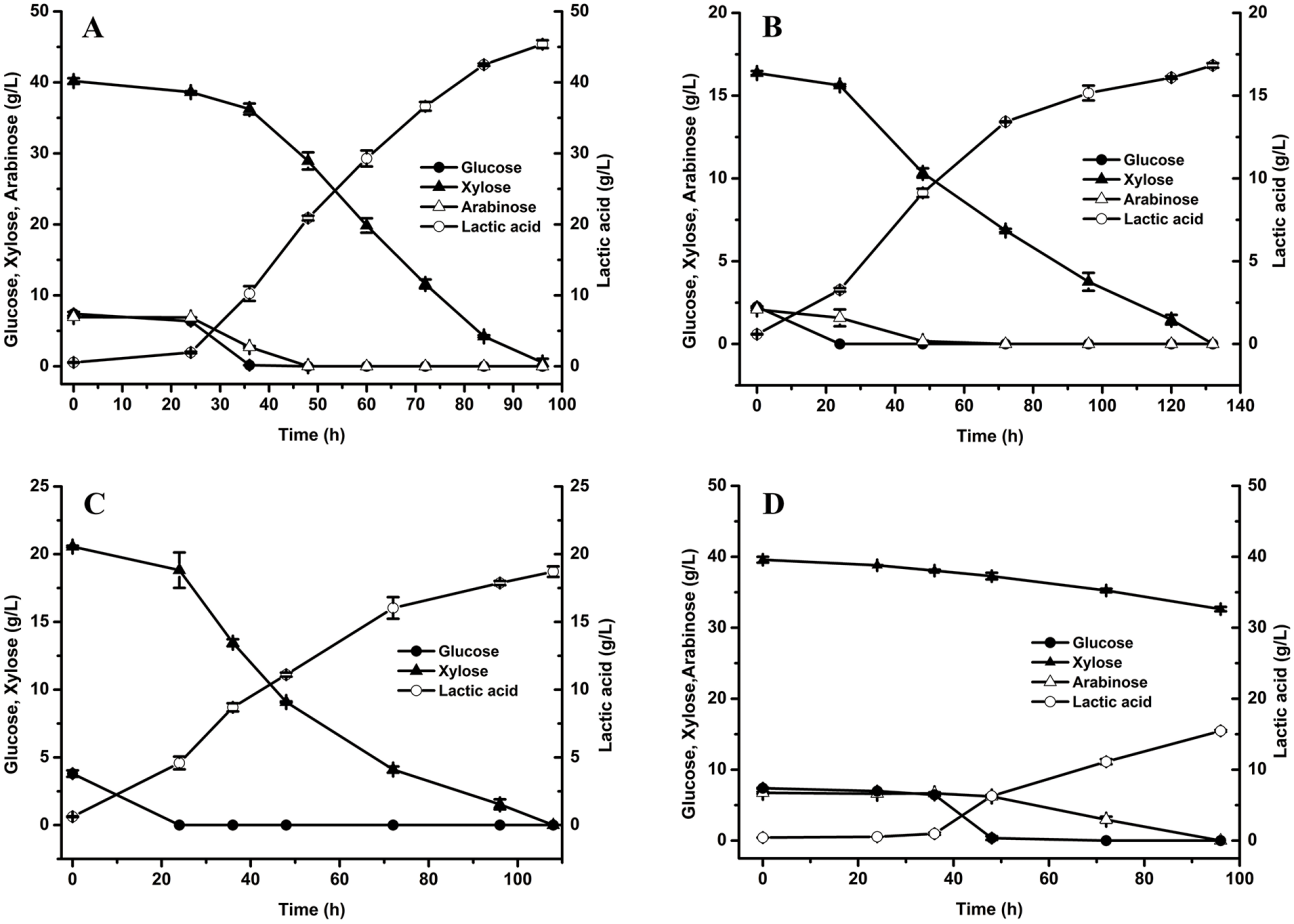
Lactic acid fermentation by *B*. *coagulans* GKN316 and *B*. *coagulans* NL01 from the three condensed hydrolysates. (**A**) *B*. *coagulans* GKN316, condensed acid-catalyzed steam-exploded hydrolysates (CASEH); (**B**) *B*. *coagulans* GKN316, condensed acid-catalyzed liquid hot water hydrolysate (CALH); (**C**) *B*. *coagulans* GKN316, condensed acid-catalyzed sulfite hydrolysate (CASH); (**D**) *B*. *coagulans* NL01, condensed acid-catalyzed steam-exploded hydrolysates (CASEH). Values are the average ± SD of three separate experiments.

Due to *B*. *coagulans* GKN316 has a better inhibitor tolerance of CDH, other actual hydrolysates, such as acid-catalyzed steam-exploded, acid-catalyzed liquid hot water or acid-catalyzed sulfite hydrolysate, were used as fermentation feedstock to evaluate the fermentability of lactic acid. Among them, CLH and CSH were further hydrolyzed by H_2_SO_4_ and then neutralized in order to obtain more xylose prior to fermentation and were referred to as condensed acid-catalyzed liquid hot water hydrolysates (CALH) and condensed acid-catalyzed sulfite hydrolysate (CASH), respectively. Table 1 shows that the hydrolysates from the different pretreatment methods had different inhibitor components and that CASEH contained the highest concentration of monosaccharides. The performances of *B*. *coagulans* GKN316 and *B*. *coagulans* NL01 were compared with respect to their ability to ferment the sugars during the anaerobic condition (Fig 2). As expected, xylose was utilized very slowly before 24 h, which indicated that a long period of adaptation was required when using these hydrolysates. When the strain adapted to the medium, a rapid consumption of xylose was observed and a high fermentation efficiency could be attained. After 96-h fermentation of CASEH, approximately 45.39 g/L lactic acid was produced, while the lactic acid yield was 83.15%. As for CALH and CASH, the sugars (xylose, glucose and arabinose) were nearly completely utilized within 132 and 108 h, respectively, and the concentration of lactic acid reached 16.83 and 18.71 g/L, respectively. However, for NL01, only CASEH could be directly fermented for lactic production, and approximately 15.47 g/L lactic acid was found in the culture after 96-h fermentation. The fermentation of CALH and CASH by NL01 failed in the present work. These results showed that our evolved strain GKN316 has a wider adaptability than the parental strain.

To date, several *B*. *coagulans* strains have been reported to produce lactic acid from lignocellulosic hydrolysates. For *B*. *coagulans* strain MXL-9, about 40 g/L lactic acid was produced from hydrolysate of corn fiber treated with dilute sulfuric acid in 72 h [19]. Our previous report showed that *B*. *coagulans* NL-CC-17 produced 23.49 g/L lactic acid in 36 h from the hydrolysate of H_2_SO_4_ catalyzed steam-exploded corn stover (xylose 22.45 g/L, glucose 3.74 g/L and arabinose 3.78 g/L), and the yield of lactic acid was 83.09% [29]. In the present study, *B*. *coagulans* GKN316 produced over 45.39 g/L lactic acid from a high concentration H_2_SO_4_ catalyzed steam-exploded hydrolysate with a yield of 83.15%. These results suggested that *B*. *coagulans* GKN316 is more adaptive to the lignocellulosic hydrolysates and is essential for economical production of lactic acid.

### Biotransformation of inhibitors by *B*. *coagulans*

The observed ability of the GKN316 strain to recover rapidly from even high concentration of hydrolysates led us to further investigate the mechanism of recovery. Previously, we showed that acetic acid and levulinic acid reduced lactic acid productivity in xylose utilization by NL01 at 15 and 1.0 g/L, respectively. Low concentrations of formic acid (<2 g/L) exerted a stimulating effect on the lactic acid production [24]. In the present work, the effect of the individual inhibitors on lactic production focuses on the furan derivatives and phenolic compounds. Furfural and HMF are commonly found in lignocellulosic hydrolysates, which inhibit the growth of many microorganisms (yeast, *Zymomonas mobilis* and *Clostridium acetobutylicum*) and also decrease yield and productivity [8,26,30,31]. As for lignin and/or its degradation products, the phenolic compounds, they are general divided into three groups: guaiacyl (G), syringyl (S) and 4-hydroxybenzyl (H) [13]. Therefore, vanillin, syringaldehyde and *p*-hydroxybenzaldehyde (pHBal) represent three different types of phenol group in lignin, which displayed high toxicity even at very low concentration [30,32,33]. Based on these considerations, two kinds of furan aldehydes (furfural and HMF) and three phenolic compounds (vanillin, syringaldehyde and pHBal) were selected to investigate the susceptibility of *B*. *coagulans*.

**Fig 3:**
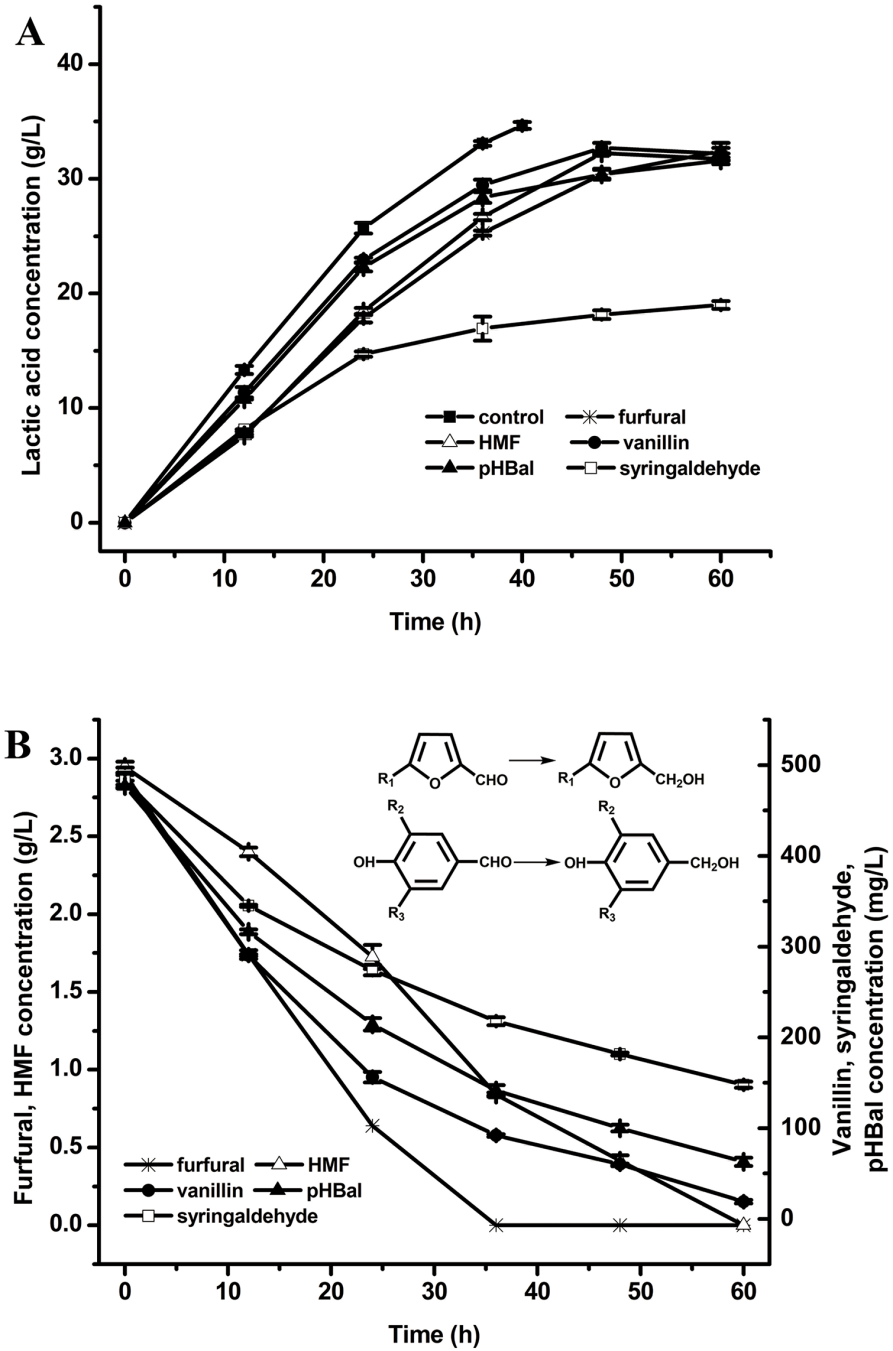
Fermentation performance of *B*. *coagulans* GKN316 in the presence of the individual inhibitors. (**A**) Lactic acid production; (**B**) Inhibitor concentration. R_1_ = H, Furfural→furfuryl alcohol; R_1_ = CH_2_OH, HMF→bis-hydroxymethylfuran (HMF alcohol); R_2_ = H, R_3_ = OCH_3_ vanillin→vanillyl alcohol; R_2_ = R_3_ = OCH_3_, syringaldehyde→syringaldehyde alcohol; R_2_ = R_3_ = H, *p*-hydroxybenzaldehyde (pHBal)→*p*-hydroxybenzyl alcohol. Values are the average ± SD of three separate experiments.

Table 2 and Fig 3A show the effect of inhibitors on growth and lactic acid production of GKN316 in xylose. The concentrations of furan aldehydes used for this experiment were 3 g/L and phenolic compounds were 0.5 g/L. The newly adapted stain was still found to be inhibited to some extent in the presence of the individual inhibitor. In the control experiment, all of the xylose was exhausted and a maximum lactic acid yield of 34.66 g/L was achieved after 40-h fermentation. The highest productivity of 0.92 g/L/h was reached at 36 h. When HMF and furfural were individually supplemented, the inhibitory effects of furfural and HMF were both observed. Compared to the control, the lactic acid productivities obviously declined, but the inhibitory effect on the final lactic acid yield only slight decreased, as shown in Fig 3A. In the presence of furfural and HMF, lactic acid productivity decreased to 0.70 or 0.74 g/L/h, respectively, after 36-h fermentation. HMF has a relatively lower inhibition than furfural. The lactic acid concentration was similar in the presence of either furfural or HMF. Moreover, the effects of the individual phenolic compounds on lactic acid fermentation were also investigated. The fermentation of xylose (40 g/L) supplemented with 0.5 g/L vanillin, syringaldehyde, or pHBal was carried out. Vanillin and pHBal demonstrated a weak negative effect on fermentation, whereas an obvious inhibition was observed toward syringaldehyde. In the presence of vanillin, pHBal, or syringaldehyde, *B*. *coagulans* GKN316 produced 32.69, 30.40, or 18.15g/L lactic acid after 48-h fermentation, respectively. The inhibition by furfural was also reported for *C*. *shehatate* and *Pichia stipitis* in which the cell growth and ethanol production were almost completely inhibited in the presence of 2 g/L furfural [14]. The fermentative activities of *Z*. *mobilis* were greatly sensitive to the presence of 0.5 g/L pHBal [14]. Compared with the reported strains, *B*. *coagulans* had better tolerance for these individual inhibitors in the hydrolysates. This is in accordance with the aldehyde tolerance of the strain MXL-9 [19]. Previous reports hold the view that the lower molecular weight compound are more toxic to microorganisms among the different phenolic compounds [13,34] and their inhibitory effects are considered relative to specific functional groups [35,36]. Our results suggested that the syringyl compound is the most toxic to *B*. *coagulans* GKN316 among the furan aldehydes and the three phenolic compounds.

**Table 2:** Cell biomass (OD_600_) of *B*. *coagulans* GKN316 in the xylose medium with different inhibitors during fermentation.

Time (h)	Control	Furfural	HMF	Vanillin	Syringaldehyde	*p*-hydroxybenzaldehyde
**0**	0.60 ± 0.01	0.60 ± 0.01	0.60 ± 0.01	0.60 ± 0.01	0.60 ± 0.01	0.60 ± 0.01
**12**	7.63 ± 0.24	5.66 ± 0.10	6.35 ± 0.27	7.10 ± 0.02	6.12 ± 0.11	6.99 ± 0.30
**24**	9.59 ± 0.03	6.47 ± 0.11	7.25 ± 0.13	7.94 ± 0.10	6.54 ± 0.14	7.53 ± 0.17
**36**	8.65 ± 0.09	6.09 ± 0.02	6.77 ± 0.36	7.72 ± 0.15	5.87 ± 0.08	7.02 ± 0.09
**48**	8.58 ± 0.31	5.98 ± 0.20	6.38 ± 0.32	7.40 ± 0.22	5.42 ± 0.16	6.62 ±0.34
**60**	8.42 ± 0.14	5.67 ± 0.13	6.30 ±0.06	7.06 ± 0.11	5.33 ± 0.24	6.51± 0.03

Values are the average ± SD of three separate experiments.

To better understand the biotransformation of the inhibitors by GKN316, the concentration of the individual inhibitors present in the culture was determined by HPLC analysis, and the constituents of the culture before and after fermentation in the presence of the different inhibitors were identified by GC—MS. Most strikingly, a clear decline in the concentration of the different inhibitors along with the fermentation time was observed for all of the examined inhibitors (Fig 3B). It was apparent that these inhibitors could be converted by GKN316, which resulted in the removal of toxic compounds and the production of less toxic compounds. Among them, 3 g/L furfural had completely disappeared after 36-h fermentation, while a small amount of HMF remained after 60-h incubation. *B*. *coagulans* GKN316 did not convert 100% of the lignin degradation products, even at 0.5 g/L, until after 60-h fermentation. Further GC—MS analysis of the extracts showed that the conversion products were confirmed as the corresponding alcohols analog of those inhibitors after fermentation (S1 Fig). The conversion mechanisms of the furan derivatives and the phenolic compounds have not been reported in lactic acid bacteria. However, it has been showed that some ethanologenic strains, either *S*. *cerevisiae* or *Z*. *mobilis*, can convert them by the reduction of the added aldehydes [25,30,34,36]. *S*. *cerevisiae* was also reported to have the ability to convert them to less harmful ones, probably due to the presence of alcohol dehydrogenases [37], aldo-keto reductases [11] and phenylacrylic acid decarboxylase [38]. Our observations confirmed that *B*. *coagulans* might have a tolerance mechanism similar to *S*. *cerevisiae* and *Z*. *mobills*, which could reduce aldehydes to the less toxic corresponding alcohols.

## Conclusion

The strain *B*. *coagulans* GKN316 had an increased inhibitor-tolerance by ARTP mutation and directed adaptation using acid hydrolysate. When using 50% CDH as the substrate, 35.37 g/L lactic acid was produced by GKN316, whereas no lactic acid was produced by NL01. Moreover, 45.39 g/L lactic acid was obtained from undetoxified CASEH in 96 h, and the yield of lactic acid was higher with 83.15%. Because of its inhibitor tolerance and its ability to fully utilize pentose sugars, GKN316 has the potential to be developed as a biocatalyst for the conversion of lignocellulosic hydrolysates into lactic acid. Tolerance studies of the individual inhibitors showed that the syringyl compound is the most toxic to *B*. *coagulans*. This is the first report showing that *B*. *coagulans* detoxifies furfural, HMF, vanillin, syringaldehyde and pHBal during fermentation *in situ* by aldehyde reduction.

## Supporting Information

S1 Fig GC chromatograms of the extracts taken with *B*. *coagulans* GKN316 at 0 h (A1, B1, C1, D1, E1) and 60 h (A2, B2, C2, D2, E2) fermentation time upon different inhibitors. Furfural (**A**), HMF (**B**), vanillin (**C**), *p*-hydroxybenzaldehyde (**D**) and syringaldehyde (**E**). MS confirmed the identity of the compound and its conversion product with > 90% confidence.
(DOCX)
