# Supplementary material for: Lactic Acid Production from Pretreated Hydrolysates of Corn Stover by a Newly Developed Bacillus coagulans Strain
Source: PLoS One. 2016 Feb 10;11(2):e0149101. doi: 10.1371/journal.pone.0149101 (PMC4749344; doi:10.1371/journal.pone.0149101)
Supplement: S1 Fig — Furfural (A), HMF (B), vanillin (C), p-hydroxybenzaldehyde (D) and syringaldehyde (E). MS confirmed the identity of the compound and its conversion product with > 90% confidence. (DOCX) [file pone.0149101.s001.docx]

**Supporting Information**

S1 Fig. GC chromatograms of the extracts taken with *B. coagulans* GKN316 at 0 h (A1, B1, C1, D1, E1) and 60 h (A2, B2, C2, D2, E2) fermentation time upon different inhibitors.

Furfural alcohol

**A1**

**A2**

Furfural

HMF alcohol?

HMF

**B1**

**B2**

Vanillin

Vanillyl alcohol

**C1**

**C2**

**D2**

*p*-hydroxybenaldehyde

*p*-hydroxybenzyl alcohol

**D1**

Syringaldehyde

Syringyl alcohol

**E1**

**E2**

**S1 Fig. GC chromatograms of the extracts taken with *B. coagulans* GKN316 at 0 h (A1, B1, C1, D1, E1) and 60 h (A2, B2, C2, D2, E2) fermentation time upon different inhibitors.** Furfural (**A**), HMF (**B**), vanillin (**C**), *p*-hydroxybenzaldehyde (**D**) and syringaldehyde (**E**). MS confirmed the identity of the compound and its conversion product with > 90% confidence.
